# Plant-Derived Purification, Chemical Synthesis, and In Vitro/In Vivo Evaluation of a Resveratrol Dimer, Viniferin, as an HCV Replication Inhibitor

**DOI:** 10.3390/v11100890

**Published:** 2019-09-23

**Authors:** Sungjin Lee, Karabasappa Mailar, Mi Il Kim, Minkyung Park, Jiseon Kim, Dal-Hee Min, Tae-Hwe Heo, Soo Kyung Bae, Wonjun Choi, Choongho Lee

**Affiliations:** 1 College of Pharmacy, Dongguk University, Goyang 10326, Korea; flatronsky@gmail.com (S.L.); kitty_1506@yahoo.com (K.M.); mi-il0826@naver.com (M.I.K.); minkyung-p@naver.com (M.P.); 2 College of Pharmacy and Integrated Research Institute of Pharmaceutical Sciences, The Catholic University of Korea, Bucheon 14662, Korea; odri1@naver.com (J.K.); thhur92@catholic.ac.kr (T.-H.H.); baesk@catholic.ac.kr (S.K.B.); 3 Department of Chemistry, Seoul National University, Seoul 08826, Korea; dalheemin@snu.ac.kr

**Keywords:** hepatitis C virus (HCV), viniferin, antiviral activity, NS3 helicase inhibitor, pharmacokinetics

## Abstract

Oligostilbenoid compounds, a group of resveratrol multimers, display several anti-microbial activities through the neutralization of cytotoxic oxidants, and by inhibiting essential host and viral enzymes. In our previous study, we identified a series of oligostilbenoid compounds as potent hepatitis C virus (HCV) replication inhibitors. In particular, vitisin B, a resveratrol tetramer, exhibited the most dramatic anti-HCV activity (EC_50_ = 6 nM and CC_50_ > 10 μM) via the disruption of the viral helicase NS3 (IC_50_ = 3 nM). However, its further development as an HCV drug candidate was halted due to its intrinsic drawbacks, such as poor stability, low water solubility, and restricted in vivo absorption. In order to overcome these limitations, we focused on (+)-ε-viniferin, a resveratrol dimer, as an alternative. We prepared three different versions of (+)-ε-viniferin, including one which was extracted from the grapevine root (EVF) and two which were chemically synthesized with either penta-acetylation (SVF-5Ac) or no acetylation (SVF) using a newly established synthesis method. We confirmed their anti-HCV replication activities and minimal cytotoxicity by using genotype 1b and 2a HCV replicon cells. Their anti-HCV replication action also translated into a significant reduction of viral protein expression. Anti-HCV NS3 helicase activity by EVF was also verified in vitro. Finally, we demonstrated that SVF has improved pharmacokinetic properties over vitisin B. Overall, the favorable antiviral and pharmacokinetic properties of these three versions of viniferin warrant their further study as members of a promising new class of anti-HCV therapeutics.

## 1. Introduction

Hepatitis C virus (HCV) is a member of the Flaviviridae family of viruses and contains a single-stranded positive RNA genome. Following the entry into a host hepatocyte, an IRES (internal ribosome entry site)-dependent translation of its RNA genome results in a long polyprotein (~3000 amino acids), which is subsequently cleaved into 10 individual viral proteins by host and virus proteases [1,2]. The E1, E2, and core viral structural proteins constitute a mature virion—however several nonstructural (NS) proteins, including NS2, NS3, NS4A, NS4B, NS5A, and NS5B, are necessary to build a functional replication complex in the endoplasmic reticulum as a self RNA copy machine [3,4,5]. Particularly, the HCV NS3 protein plays two essential roles in the viral life cycle. First, coordination between its N-terminally encoded protease activity and a viral scaffold protein (NS4A) is required for the efficient cleavage of a viral polyprotein. Second, its C-terminally encoded helicase activity plays an essential role in the HCV life cycle. The NS3 helicase assists in HCV RNA replication by resolving double-stranded RNA intermediates formed during viral RNA replication [6]. However, the development of HCV NS3 helicase inhibitors has been difficult due to their homology with host helicase proteins.

Approximately 170 million people are estimated to be infected with HCV worldwide [7]. Once infected, ~70–80% of HCV-positive carriers develop a chronic infection with a high chance of developing chronic liver diseases (e.g., liver cirrhosis and hepatocellular carcinoma) ~ 20 years after infection [8,9]. HCV infection is the primary cause of liver transplantation in the United States, accounting for approximately 40~45% of all liver transplants [10]. Traditionally, the treatment for HCV infection has relied on a combination of pegylated interferon-α and ribavirin. Recently, different combinations of direct-acting antiviral drugs with improved antiviral efficacy have emerged. They include glecaprevir (NS3/4A protease inhibitor)/pibrentasvir (NS5A inhibitor), sofosbuvir (NS5B RNA polymerase inhibitor)/ledipasvir (NS5A inhibitor), sofosbuvir/daclatasvir (NA5A inhibitor), sofosbuvir/simeprevir (NS3/4A protease inhibitor), sofosbuvir/velpatasvir (NS5A inhibitor), and elbasvir (NS5A inhibitor)/grazoprevir (NS3/4A protease inhibitor) [11]. Depending on the HCV genotype, the liver condition of the patient (cirrhosis), HIV coinfection status, and the patient’s ethnicity, a specific type and duration of anti-HCV treatment regimens can be customized [12]. This leads to remarkable clearance rate of approximately 90% among all chronic HCV cases with the new combination treatment regimen [13]. However, in spite of this progress, morbidity and mortality associated with HCV infection still constitute a large burden on the health care system of affected countries.

**Figure 1:**
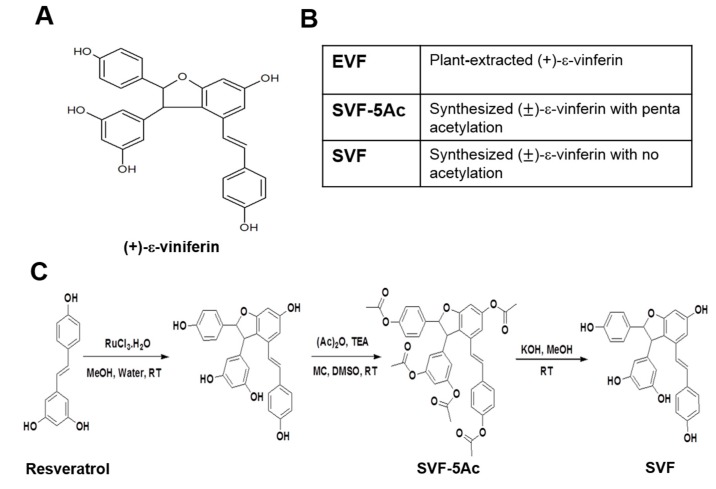
(**A**) The chemical structure of (+)-ε-viniferin (**B**) three different versions of (+)-ε-viniferin, including one which was extracted from the grapevine root (EVF) and two which were chemically synthesized with either penta-acetylation (SVF-5Ac) or no acetylation (SVF) (**C**) The organic synthesis process of SVF-5Ac and SVF from resveratrol.

In our previous study, we observed the inhibitory activity of grapevine root extract against HCV replication through cell-based screening of the Asian herbal plants extract library [14]. Especially, a group of oligostilbenoid compounds such as ampelopsin A, (+)-ε-vinifirin, wilsonol C, vitisin A, and vitisin B were purified from grapevine root. Among them, we identified vitisin B as the most potent HCV replication suppressor, and its mechanism of action relies on inhibiting the HCV NS3 helicase. However, an undesirable pharmacokinetic property of vitisin, and the difficulties associated with synthesizing a compound of its size (molecular weight 906), precluded its further development. Instead, we pursued one of our previously identified anti-HCV replication inhibitors, (+)-ε-viniferin (Figure 1A), as an alternative candidate, and established a new synthesis methodology. In this study, we prepared three versions of viniferin—one was a plant extract (EVF) and two were chemically synthesized with either penta-acetylations (SVF-5Ac) or no acetylation (SVF) (Figure 1B). Their anti-HCV replication activities in HCV genotype 1b and 2a replicons were compared in parallel. The suppression of HCV NS3 helicase activity by EVF was further studied. Finally, the pharmacokinetic properties of SVF upon oral and intraperitoneal administration were examined in a mouse model.

## 2. Materials and Methods

### 2.1. Preparation of EVF from Vitis Vinifera

The roots of *Vitis vinifera* (600 g) were pulverized, mixed with ethanol (5 L), and evaporated under reduced pressure to create an ethanol extract (43 g). The ethanol extract was suspended in water and successively partitioned with n-hexane, ethyl acetate, and n-butanol. The ethyl acetate soluble extract (EA, 14.1 g) was subjected to silica gel column chromatography (CC) where a chloroform–methanol mixture [chloroform–methanol; 50:1 (Fr. EA-A), 20:1 (Fr. EA-B), 10:1 (Fr. EA-C), 5:1 (Fr. EA-D), 2:1 (Fr. EA-E), and 1:1 (Fr. EA-F)] was used during elution. Fr. EA-B (6.3 g) was chromatographed on a silica gel CC [chloroform-methanol, 25:1 (*v/v*)] to give Fr. EA-Ba – EA-Bh. The Fr. EA-Bd (150 mg) was subjected to flow-rate gradient HPCCC using a two-phase solvent system composed of n-hexane-ethyl acetate-methanol-water [4:8:4:10 (*v/v*), reversed-phase mode, mobile phase flow rate: 4 mL/min in 0–70 min, 8 mL/min in 70–250 min] to yield 8.9 mg of EVF. The structure of EVF was identified by comparing its ^1^H NMR, ^13^C NMR, and Q-TOF MS spectroscopic data [15,16,17,18].

### 2.2. Organic Synthesis of SVF-5Ac and SVF

All chemicals were purchased from commercial sources and were used without further purification. Product purification occurred via flash column chromatography, and a TLC analysis was performed on commercial plates coated with silica gel 60 F_254_. Spots on the TLC plates were visualized by UV radiation. HRMS was recorded on a Water’s Q-TOF mass spectrometer. ^1^H and ^13^C NMR spectral analyses were performed on a Varian spectrometer at 400 MHz using TMS as an internal standard. Coupling constants (J) are reported in Hz. Standard abbreviations s, d, t, and m refer to singlet, doublet, triplet, and multiplet, respectively. The purity of the products was analyzed by reversed-phase high-pressure liquid chromatography (RP-HPLC), which was performed on a Waters Corp. HPLC system equipped with a UV detector set at 254 nm. The mobile phases consisted of A) H_2_O containing 0.05% TFA and B) CH_3_CN. The HPLC employed a YMC Hydrosphere C18 (HS-302) column (5μ particle size, 12 nM pore size), 4.6 mm dia. × 150 mm with a flow rate of 1.0 mL/min. Compound purity was assessed using a gradient of 25 % B to 100 % B in 35 min.

To synthesise SVF-5Ac, a stirred solution of resveratrol (1.0 g, 4.38 mmol) in methanol/water (10:1, 11 mL) and ruthenium (III) chloride hydrate (1.09 g, 5.26 mmol) was added at 0 °C. The reaction mass was stirred at 35 °C for 3 h. After the removal of volatiles in a vacuum, the crude residue was dissolved in ethyl acetate (200 mL) and washed with sat. brine. The organic layer was dried over anhydrous MgSO_4_, filtered, and evaporated. The residue was purified by silica gel column chromatography (0–20% acetone in methylene chloride) to give an SVF-5Ac-enriched product (340 mg) and unreacted resveratrol (350 mg, 1.53 mmol). The SVF-5Ac-enriched product was dissolved in dichloromethane (20 mL) and DMSO (1 mL). Triethylamine (2.8 mL, 17.8 mmol) and Ac_2_O (1.36 mL, 14.4 mmol) were added at 0 °C and then stirred at room temperature for 5 h. The reaction mixture was diluted with dichloromethane (100 mL) and then washed with sat. NaHCO_3_ and sat. brine, sequentially. The organic layer was dried over anhydrous MgSO_4_, filtered, and evaporated. The crude product was purified by silica gel column chromatography (15%–25% ethyl acetate in hexanes) to render SVF-5Ac (0.18 g, 18% in two steps) as an off-white solid: ^1^H NMR (400 MHz, CDCl_3_) δ 7.3 (d, J = 8.8 Hz, 2 H), 7.18 (d, J = 8.4 Hz, 2 H), 7.1 (d, J = 8.4 Hz, 2 H), 6.99 (d, J = 8.4 Hz, 2 H), 6.94 (s, 1 H), 6.9 (s, 1 H), 6.89 (d, J = 16.8 Hz, 1 H), 6.85 (s, 2 H), 6.64 (s, 1 H), 6.55 (d, J = 16 Hz, 1 H), 5.6 (d, J = 6.8 Hz, 1 H), 4.6 (d, J = 6.4 Hz, 1 H), 2.33 (s, 3 H), 2.29 (s, 3 H), and 2.26 (bs, 9 H); ^13^C NMR (100 MHz, CD_3_OD) δ 169.4-168.7, 160.8, 152-150.3, 144.3, 138, 135.2, 134.3, 130.3, 127.7, 126.6, 123.9, 123.8, 121.9, 121.7, 118.5, 114.8, 110.6, 102.8, 92.6, 56.6, and 21.2-21; IR (neat) 1741.5, 1588.1, 1369.8, 1185.3, 1122.9, and 1013.7 cm^-1^; HPLC: 97.9% (retention time, 21.5 min); HRMS (ESI) m/z calcd for C_38_H_33_O_11_ [M+H]^+^: 665.2023, found: 665.2023.

For the synthesis of SVF, KOH (25 mg, 0.44 mmol) was added at room temperature to a stirred solution of SVF-5Ac 1 (50 mg, 0.075 mmol) in methanol (6 mL). The mixture was stirred for 30 min. After the methanol was removed under a vacuum, the crude residue was dissolved in ethyl acetate (25 mL) and 1 N HCl (10 mL). The partitioned organic layer was dried over anhydrous MgSO_4_, filtered, and evaporated. The crude residue was purified by recrystallization (acetone/dichloromethane/hexanes to remove non-polar impurities and then acetone/dichloromethane to remove polar impurities) to yield SVF (30 mg, 88%) as a pale yellow solid: ^1^H NMR (400 MHz, CD_3_OD) δ 7.14 (d, J = 8.8 Hz, 2 H), 7.04 (d, J = 8 Hz, 2 H), 6.83 (d, J = 16.4 Hz, 1 H), 6.75 (d, J = 8.4 Hz, 2 H), 6.65 (d, J = 8.4 Hz, 2 H), 6.62 (d, J = 1.6 Hz, 1 H), 6.58 (d, J = 16.4 Hz, 1 H), 6.24 (d, J = 1.6 Hz, 1 H), 6.17 (d, J = 2 Hz, 1 H), 6.16 (bs, 2 H), 5.37 (d, J = 6.8 Hz, 1 H), and 4.35 (d, J = 6.8 Hz, 1 H); ^13^C NMR (100 MHz, CD_3_OD) δ 161.3, 158.3-156.9, 145.9, 135.5, 132.4, 128.9, 127.3, 126.7, 122.2, 118.6, 114.9, 106, 102.9, 100.7, 95.4, 93.4, and 56.8; IR (neat) 3311.3, 1590.7, 1510.1, 1440, 1237.2, 1148.9, and 1115.1 cm^-1^; HPLC: 95.13 (retention time, 9.1 min); HRMS (ESI) m/z calcd for C_28_H_23_O_6_ [M+H]^+^: 455.1495, found: 455.1487.

### 2.3. Cell Culture

Huh7.5-based HCV replicon cells were maintained in Dulbecco’s modified Eagle’s medium (DMEM, Hyclone) supplemented with 1% L-glutamine (Hyclone, Logan, UT, USA), 1% penicillin-streptomycin (Hyclone, Logan, UT, USA), 1% non-essential amino acid (Hyclone, Logan, UT, USA), and 10% fetal bovine serum (JR Scientific, Woodland, CA, USA).

### 2.4. Plasmids

Rluc-J6/JFH1 (FL-J6/JFH-5′C19Rluc2AUbi) [19] is a monocistronic, full-length HCV genome that expresses a renilla luciferase and infectious genotype 2a HCV genome J6/JFH1 [20]. Bart79I is a high-efficiency bicistronic subgenomic replicon of HCV derived from an HCV genotype 1b Con1 sequence that harbors the neomycin phosphotransferase gene in the first cistron and the HCV nonstructural proteins in the second cistron under the translational control of an EMCV internal ribosome entry site (IRES) [21]. This plasmid also has an adaptive mutation (S2204I) in NS5A, which increases replication efficiency. Bart79I-YFP was made by the removal of amino acids 2209 to 2254 in Bart79I and the insertion of a PCR-amplified YFP sequence from pEFYP-C1 (Clonetech, Mountain View, USA) using a flanking NotI site for direct cloning into NS5A of Bart79I. FL-J6/JFH-5′C19Rluc2AUbi and Bart79I were gifts from Dr. Charles Rice at Rockefeller University. Cell-culture produced infectious HCV (HCVcc) expressing an HCV NS5A-GFP fusion protein [22].

### 2.5. In Vitro Transcription for the Production of HCV RNA Genomes

HCV RNA genomes were produced via in vitro transcription. [23]. Briefly, wild type Bart79I, J6/JFH1, or RLuc-J6/JFH1 plasmids were linearized by ScaI (NEB) digestion for Bart79I or XbaI (NEB) digestion for J6/JFH1. The T7 promoter-driven in vitro transcription was performed on the digested plasmid to produce the wild type HCV RNA genomes using a MEGAscript kit (Ambion, Waltham, MA, USA).

### 2.6. Western Blot Analysis

Either Huh7.5-J6/JFH1 or Bart79I replicon cells were plated onto a six well plate (Costar) and supplemented with DMSO, EVF, SVF, or SVF-5Ac at indicated concentrations. At 120 h after incubation, whole-cell extracts were prepared in RIPA buffer (150 mM NaCl, 1% Triton X-100, 1% deoxycholic acid sodium salt, 0.1% sodium dodecyl sulfate, 50 mM Tris-HCl, 2 mM EDTA, pH 7.5; genDEPOT, Katy, TX, USA) containing a cocktail of Complete protease inhibitors (Roche, Basel, Switerland) and quantitated by the Bradford assay (Bio-Rad, Hercules, CA, USA). Equal amounts of protein were electrophoresed on an SDS–polyacrylamide gel, subsequently transferred to a polyvinylidene difluoride membrane (Immobilon-P; Millipore, Burlington, **VT,** USA), and probed with a mouse anti-Core antibody or an anti-NS5A monoclonal antibody (1:1000, 1868 for Core, 1:1000, 1847 for NS3 1:1000, and 1877 for NS5A; Virostat, Westbrook, ME, USA). A time response curve was also produced by performing similar Western blot analyses at 24, 48, and 72 h after treating cells with DMSO and 10 µM of EVF, SVF, or SVF-5Ac.

### 2.7. Quantitative Real-Time RT-PCR (qRT-PCR) Analysis

Either Huh7.5-J6/JFH1 or Bart79I replicon cells were plated onto a six well plate (Costar, New York, NY, USA) and supplemented with DMSO, EVF, SVF, or SVF-5Ac with indicated concentrations. Three days after incubation, total cellular RNA was extracted using the RNeasy^®^ mini kit (Qiagen, Venlo, The Netherlands) in accordance with the manufacturer’s instructions. The yield of the extracted RNA was assessed spectrophotometrically. The expression of HCV subgenomic RNA and cellular RNA was measured by quantitative real-time reverse-transcription polymerase chain reaction (qRT-PCR) analysis. The qRT-PCR analysis was performed using a CFX384 qRT-PCR system (Bio-Red, Hercules, CA, USA), and the amplification program included 40 cycles at 94°C for 10 s (denaturing), 55 °C for 15 s (annealing), and 72 °C for 30 s (extension). Each sample was normalized by the endogenous reference gene glyceraldehydes-3-phosphate dehydrogenase (GAPDH). The cDNA quantification was performed using the CFX384 real-time PCR detection system (Bio-Rad). The primers used in the qRT-PCR reactions were as follows. FW-J6/JFH1-CTCCGCCATGAATCACTC, RV-J6/JFH1-ACGACACTCATACTAACGC, FW-Bart79I-AGAGCCATAGTGGTCT, RV-Bart79I-CCAAATCTCCAGGCATTGAGC, FW-GAPDH-TGGTCTCCTCTGACTTCA, and RV-GAPDH-CGTTGTCATACCAGGAAATG. A time response curve was also produced by measuring renilla luciferase activity, as well as cell viability at 24, 48, and 72 h after treating HCV RNA-transfected cells with DMSO and 10 µM of VF.

### 2.8. Graphene Oxide (GO)-Based NS3 Helicase Inhibition Assay

The helicase assay was performed using a graphene oxide-based NS3 helicase inhibition assay. [24]. Briefly, a double-stranded DNA substrate of the HCV NS3 helicase was prepared by mixing 2 μL of a 10 μM Cy5-labeled DNA strand (5′-Cy5-TGG CGA CGG CAG CGA GGC AGA GGA GCA GAG GGA GCA-3′ Genotech, Daejeon, Korea) with a two-fold excess of the complementary DNA strand (5′-GCC TCG CTG CCG TCG CCA-3′, Genotech, Daejeon, Korea) with a subsequent annealing process at 95 ℃ for 5 min in annealing buffer solution (50 mM Tris-HCl, pH 8.0, Fischer) and 50 mM NaCl (Junsei, Tokyo, Japan)]. A helicase substrate cocktail was prepared in a 96 well, black-walled plate by mixing 20 nM annealed Cy5-labeled dsDNA substrate with a 0.3 M EDTA (pH 8.0) solution (Bio-Rad) and 20 mM ATP (Sigma, St.Lous, USA) in a 1× reaction buffer [50 mM Tris-HCl (pH 8.0), 50 mM NaCl, 10% glycerol (Bio Basic Inc., ON, Canada), and 0.65 mM MgCl_2_ (Junsei)] in total volume of 30 μL. To the prepared substrate cocktail, 15 μL of GO solution (20 μg/mL in 1× reaction buffer) was added, and the mixture was incubated for 10 min at room temperature. Then, 5 μL of HCV NS3 helicase in 1× reaction buffer with various concentrations of EVF were added to the mixture of substrate cocktail and GO solution after 10 min of pre-incubation. The final concentrations of the key components in the reaction mixture were 10 nM (Cy5-dsDNA substrate), 5 μg/mL of GO, and 2 nM of HCV NS3 helicase in a total volume of 60 μL with 1× reaction buffer. Finally, the unwinding activity and inhibition efficiency of the helicase was measured via fluorescence intensity at Ex/Em = 650/675 nm using a SynergyMx (BioTek, Winooski, VT, USA) fluorometer.

### 2.9. In Vivo Pharmacokinetic Study of SVF

Institute of Cancer Research (ICR) mice (male, 9 weeks of age, 35‒40 g) were obtained from Orient Bio (Sungnam, Korea), and were acclimatized in the animal research facility of the Catholic University of Korea (CUK) for at least one week. All animal experiments were performed in accordance with the protocols approved by the Institutional Animal Care and Use Committee of CUK. SVF was administered orally (50 mg/kg, *n* = 4) or intraperitoneally (7.5 mg/kg, *n* = 4). SVF was dissolved with 1.5% (*v/v*) dimethylsulfoxide in distilled water and each mouse was administered a volume of 0.7 mL/100 g. Blood samples (approximately 20 μL) were serially collected using 20 μL heparinized microcapillaries and a micropipette after a slight incision of the lateral tail vein, as previously described [25] at 5, 15, 30, 60, 120, 240, 360, and 480 min after SVF dosing. The blood samples were transferred into 0.2 mL microcentrifuge tubes and immediately centrifuged at 15,000× *g* for 5 min at 4 °C to separate the plasma (8 μL), which was stored at –80 °C until analysis. The detailed analytical conditions and assay validation parameters were previously reported [25]. The pharmacokinetic parameters of SVF were calculated by the non-compartmental method using WinNonlin software (Pharsight Corp., Version 5.0.1).

### 2.10. Statistical Analysis

Values in the graphs represent the mean and standard deviations of representative experiments performed in triplicate or quadruplicate using Prism v5.0c software. Calculated *p*-values from the Student’s *t*-test less than 0.05, when compared with a control, were considered statistically significant. A single asterisk (*) indicates that the *p*-value is between 0.1 and 0.5. A double asterisk (**) indicates that a *p*-value is between 0.1 and 0.01. A triple asterisk (***) indicates that the *p*-value is less than 0.01. The resulting data were fit to the Hill equation using the Prism v5.0c software to calculate EC_50_ and CC_50_ values. We recalculated all the half-life values (T_1/2_) by using the following equation for one phase decay: Y = (Y0 - Plateau)*exp(-K*X) + Plateau, where X = Time, Y = RNA levels, starting at Y0 and decaying (with one phase) down to plateau; Y0 and plateau have same units as Y; K = Rate constant equal to the reciprocal of the X-axis units [26].

## 3. Results

### 3.1. Isolation of Plant-Derived EVF and Organic Synthesis of SVF-5Ac and SVF

In previous studies, we isolated five resveratrol oligomers, such as ampelopsin A, (+)-ε-viniferin, wilsonol C, vitisin A, and vitisin B from grapevine roots and confirmed their inhibitory action against HCV replication. Furthermore, we chose vitisin B since it was the most potent inhibitor of HCV replication. However, despite its significant ability to prevent HCV replication in vitro, it showed low bioavailability due to its relatively large molecular size and low water solubility [14]. Although EVF exhibited approximately 30-fold less antiviral activity than vitisin B, its smaller size (half of vitisin B) was expected to show better pharmacokinetic properties when compared with vitisin B.

First, we isolated pure (+)-ε-vinifirin from grapevine roots using a previously published purification protocol and named this compound as plant-extracted (+)-ε-viniferin (EVF) (Figure 1A) [14]. However, scaling-up the purification of EVF was impossible due to the scarcity of the plant source. Thus, we decided to perform a chemical synthesis of vinifirin instead. According to the literature, the synthesis of (+)-ε-viniferin can be performed through an oxidative coupling reaction of resveratrol [27]. However, reacting resveratrol with FeCl_3_·6H_2_O as the oxidizing agent produced a very low and inconsistent yield of product, as well as a large quantity of inseparable adducts (data not shown). To overcome these drawbacks, we employed diverse oxidizing agents and obtained improved results when ruthenium chloride (RuCl_3_·H_2_O) was used as the oxidant (Figure 1C). Even though the reaction yield was only 21% of the starting material, the formation of (±)- -viniferin (SVF) was sufficient and reproducible. The isolation of the product in a pure state was problematic because of the polarity caused by the presence of many free hydroxyl groups. Hence, impure SVF resulting from oxidative dimerization was acetylated to yield penta-acetylated (±)- -viniferin (SVF-5Ac) [28], which was easily purified by flash column chromatography. Subsequent deacetylation of SVF-5Ac with KOH in methanol yielded the final product [27] without column chromatography purification and with excellent yield (88%) (Figure 1C). The newly developed method was suitable for the large scale synthesis of SVF [27].

### 3.2. Inhibition of HCV Replication by EVF, SVF-5Ac, and SVF

**Figure 2:**
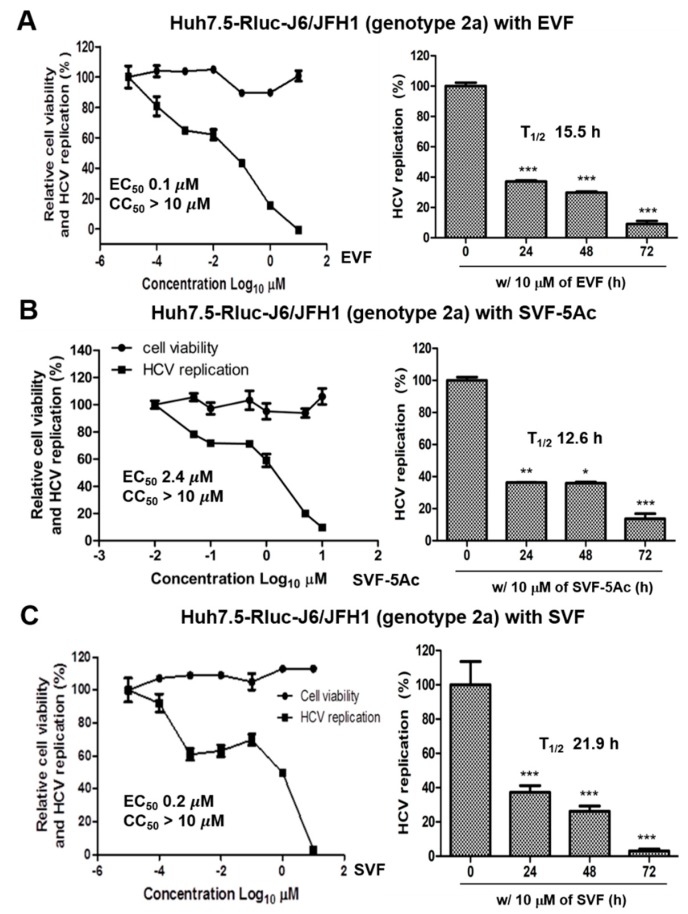
The concentration and time-dependent effects of (**A**) EVF, (**B**) SVF-5Ac, and (**C**) SVF on genotype 2a HCV replication and cell viability in Rluc-J6/JFH1 RNA-transfected Huh7.5 cells. HCV replication and cell viability were measured by luciferase and MTT assays in renilla luciferase-J6/JFH1 RNA-transfected Huh7.5 cells after treating them with an indicated compound for 72 h. EC_50_ is the concentration required for 50% inhibition of HCV replication. CC_50_ is the concentration required for 50% inhibition of cell viability. T_1/2_ is the time required for 50% inhibition of HCV replication at 10 μM of the indicated compound. A single asterisk (*) indicates that the *p*-value is between 0.1 and 0.5. A double asterisk (**) indicates that a *p*-value is between 0.1 and 0.01. A triple asterisk (***) indicates that the *p*-value is less than 0.01.

In order to study the effects of EVF, SVF-5Ac, and SVF on HCV replication, we transfected huh7.5 hepatocarcinoma cells with in vitro transcribed renilla luciferase-linked genotype 2a J6/JFH1 RNAs [19] and treated HCV RNA-transfected Huh7.5 cells with either EVF, SVF-5Ac, or SVF for 72 h. Then, the luciferase activity was measured as a surrogate for HCV RNA replication together with cell viability. EVF exhibited the most potent antiviral activity with little cytotoxicity (EC_50_ = 0.1 μM and CC_50_ > 10 μM) followed by SVF-5Ac (EC_50_ = 2.37 μM and CC_50_ > 10 μM) and SVF (EC_50_ = 0.2 μM and CC_50_ > 10 μM) (Figure 2). A time-dependent reduction in HCV RNA genome replication was also confirmed with the application of 10 μM of either EVF, SVF-5Ac, or SVF (Figure 2). Their half-life (T_1/2_) values (i.e., the time required for a 50% reduction of HCV RNA genome replication in the presence of the compound at 10 μM) were comparable to each other (15.5 h for EVF, 12.6 h for SVF-5Ac, and 21.9 h for SVF).

**Figure 3:**
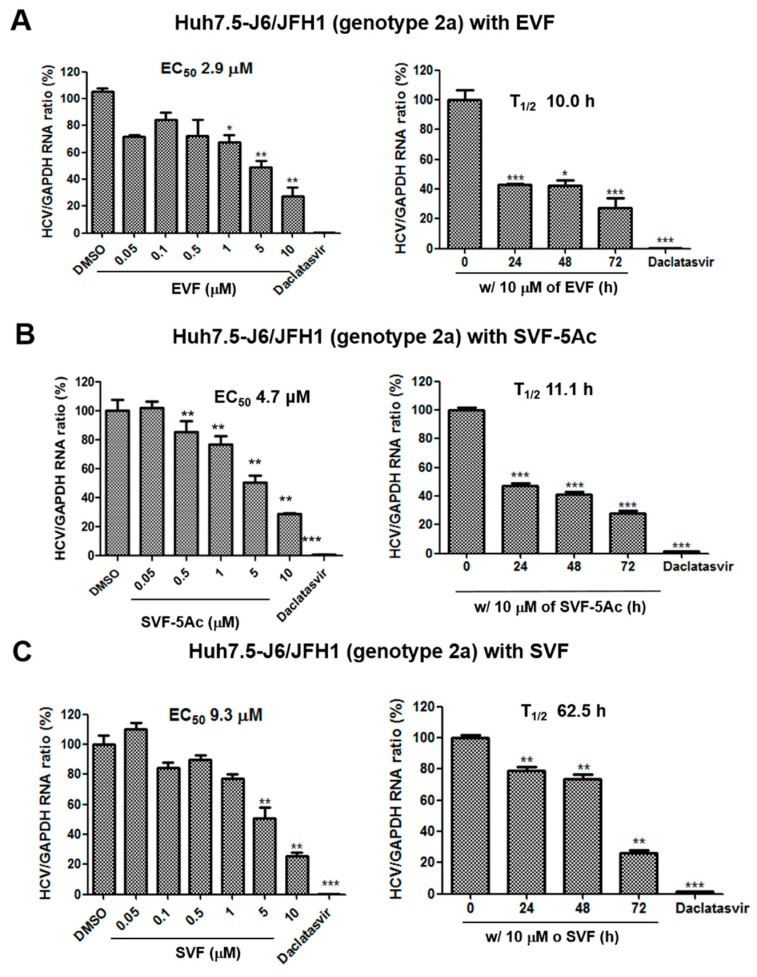
The concentration- and time-dependent effects of (A) EVF, (B) SVF-5Ac, and (C) SVF on genotype 2a HCV replication and cell viability in J6/JFH1 RNA-transfected Huh7.5 cells. Their impacts on HCV replication and cell viability were measured with real-time RT-PCR and MTT assays in J6/JFH1 RNA-transfected Huh7.5 cells. EC_50_ is the concentration required for 50% inhibition of HCV replication. T_1/2_ is the time required for 50% inhibition of HCV replication at the concentration of 10 μM. 1 nM of daclatasvir, an NS5A inhibitor, was used as a positive control. A single asterisk (*) indicates that the *p*-value is between 0.1 and 0.5. A double asterisk (**) indicates that a *p*-value is between 0.1 and 0.01. A triple asterisk (***) indicates that the *p*-value is less than 0.01.

**Figure 4:**
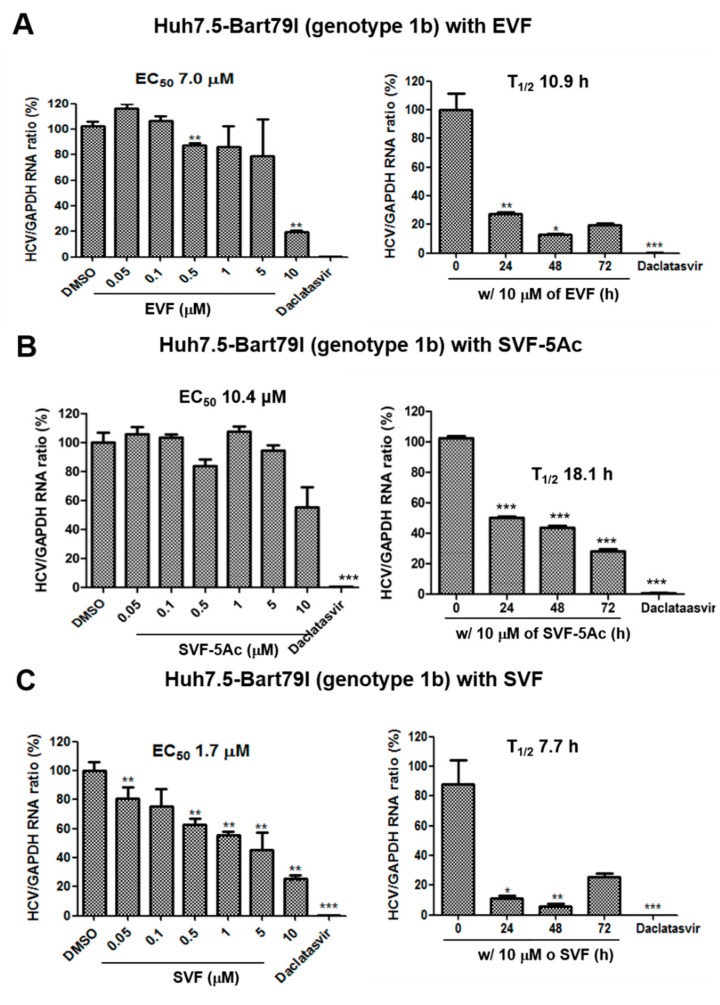
The concentration- and time-dependent effects of (**A**) EVF, (**B**) SVF-5Ac, and (**C**) SVF on genotype 1b HCV replication and cell viability in Bart79I RNA-transfected Huh7.5 cells. Their impacts on HCV replication and cell viability were measured with real-time RT-PCR and MTT assays in Bart79I RNA-transfected Huh7.5 cells. EC_50_ is the concentration required for 50% inhibition of HCV replication. T_1/2_ is the time required for 50% inhibition of HCV replication at the concentration of 10 μM. 1 nM of daclatasvir, an NS5A inhibitor, was used as a positive control. A single asterisk (*) indicates that the *p*-value is between 0.1 and 0.5. A double asterisk (**) indicates that a *p*-value is between 0.1 and 0.01. A triple asterisk (***) indicates that the *p*-value is less than 0.01.

To rule out the possibility of an artificial effect of the inserted renilla luciferase on HCV replication, we tested its impact on HCV replication with real-time RT-PCR analysis. We first transfected Huh7.5 cells with full-length infectious J6/JFH1 RNAs (Huh7.5-J6/JFH1) (genotype 2a) [20], and then we treated the transfected cells with an increasing concentration of either EVF, SVF-5Ac, or SVF for 72 h. In addition, we also treated the transfected cells with 10 μM of either EVF, SVF-5Ac, or SVF for an increasing period of time to study any time-dependent effects on HCV replication. As shown in Figure 3, real-time RT-PCR analyses confirmed that the three versions of vinifirin exhibited dose- and time-dependent inhibition of HCV replication. Their EC_50_ and T_1/2_ values were: EVF (EC_50_ = 2.9 μM and T_1/2_ = 10.0 h) (Figure 3A), SVF-5Ac (EC_50_ = 4.7 μM and T_1/2_ = 11.1 h) (Figure 3B), and SVF (EC_50_ = 9.3 μM and T_1/2_ = 62.5 h) (Figure 3C). Their overall antiviral potency was reduced in Huh7.5 cells maintaining a genotype 2a HCV replicon without a renilla luciferase. When we performed a similar experiment using sub-genomic genotype 1b HCV replicon cells (Huh7.5-Bart79I) harboring a neomycin-resistant gene, we also confirmed their anti-HCV replication activities in a dose- and time-dependent fashion (Figure 4). In particular, SVF displayed the most potent antiviral activity in genotype 1b HCV replicon cells with an EC_50_ value of 1.7 μM (Figure 4C). The EC_50_ and T_1/2_ values for EVF and SVF-5As were: EVF (EC_50_ = 7.0 μM and T_1/2_ = 10.9 h) (Figure 4A), SVF-5Ac (EC_50_ = 10.4 μM and T_1/2_ = 18.1 h) (Figure 4B). These data indicate an efficient inhibition of genotype 2a and 1b HCV replication by all three versions of viniferin with minimal cytotoxicity.

### 3.3. Inhibition of HCV Protein Expression by EVF, SVF-5Ac, and SVF

**Figure 5:**
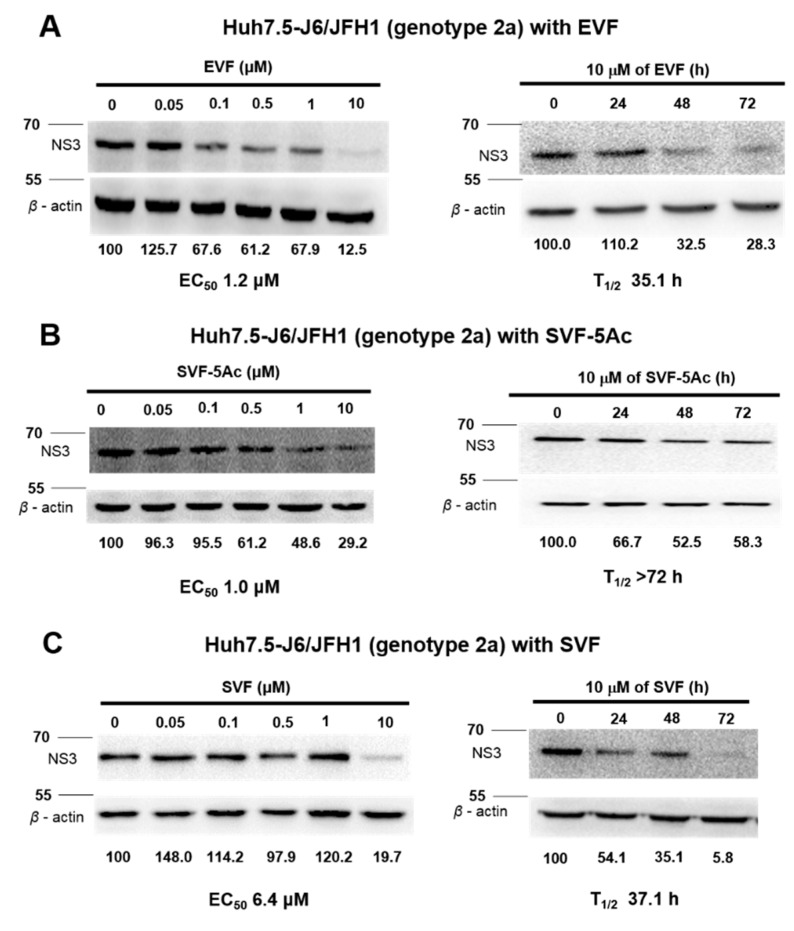
The concentration- and time-dependent inhibitory effects of (**A**) EVF, (**B**) SVF-5Ac, and (**C**) SVF on genotype 2a HCV protein expression in J6/JFH1 RNA-transfected Huh7.5 cells. Their impacts on HCV protein expression were measured by Western blot analysis of the HCV NS3 protein in J6/JFH1 RNA-transfected Huh7.5 cells. EC_50_ is the concentration required for 50% inhibition of HCV NS3 protein expression. T_1/2_ is the time required for 50% inhibition of HCV NS3 protein expression at the concentration of 10 μM. Positions of 55 and 70 kd protein size markers were indicated with lines.

**Figure 6:**
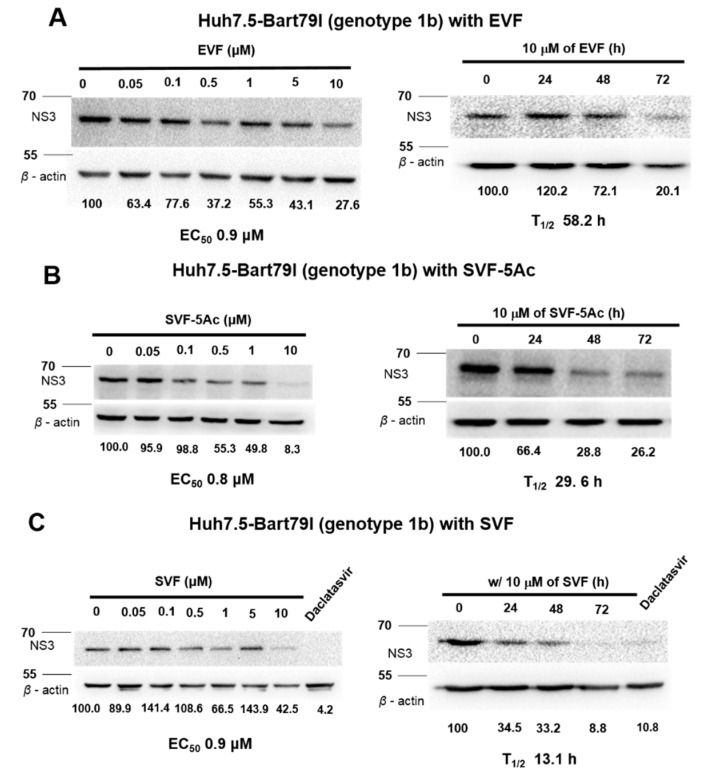
The concentration- and time-dependent effects of (**A**) EVF, (**B**) SVF-5Ac, and (**C**) SVF on genotype 1b HCV protein expression in Bart79I RNA-transfected Huh7.5 cells. Their impacts on HCV protein expression were measured by Western blot analysis in Bart79I RNA-transfected Huh7.5 cells. EC_50_ is the concentration required for 50% inhibition of HCV protein expression. 1 nM of daclatasvir, an NS5A inhibitor, was used as a positive control. Positions of 55 and 70 kd protein size markers were indicated with lines.

The inhibition of RNA virus genome replication results in the suppression of viral protein expression. Thus, we wanted to determine whether a decrease in viral protein expression occurs as a result of the inhibitory effects of EVF, SVF-5Ac, and SVF on HCV replication. For this purpose, we treated J6/JFH1 RNA-transfected Huh7.5 cells with an increasing concentration of either EVF, SVF-5Ac, or SVF for 120 h. As shown in Figure 5, all three compounds were able to reduce HCV NS3 protein production in a dose-dependent manner. The concentrations required for a 50% reduction of the NS3 protein (EC_50_) were: 1.23 μM for EVF, 1.0 μM for SVF-5Ac, and 6.4 μM for SVF. Furthermore, using a fixed concentration (10 μM) of each compound for a different incubation time allowed us to calculate the half-life (T_1/2_) required for a 50% reduction of the HCV NS3 protein. Their T_1/2_ values were: 35.1 h for EVF, >72 h for SVF-5Ac, and 37.1 h by SVF (Figure 5). In addition, genotype 1b subgenomic Bart79I cells were also used to evaluate the dose- and time-dependent effects of EVF, SVF-5Ac, and SVF on viral protein levels. EVF, SFV-5Ac, and SVF all showed a more dramatic reduction in the HCV NS5A protein level in Bart79I RNA-transfected cells than J6/JFH1-transfected cells (Figure 6). EC_50_ and T_1/2_ values, determined by measuring the NS3 protein level in Bart79I-transfected Huh7.5 cells, were: EVF (EC_50_ = 0.9 μM and T_1/2_ = 58.2 h) (Figure 6A), SVF-5Ac (EC_50_ = 0.8 μM and T_1/2_ = 29.6 h) (Figure 6B), and SVF (EC_50_ = 0.9 μM and T_1/2_ = 13.1 h) (Figure 6C). Collectively, these data suggest that all three versions of vinifirin can reduce HCV protein production by inhibiting viral genome replication. All the EC_50_ values for EVF, SVF-5Ac, and SVF, which were determined by using genotype 2a and 1b luciferase reporter assay, RT-PCR, and Western blot analyses are summarized in Table 1.

### 3.4. Inhibition of Genotype 1b HCV NS3 Helicase by EVF

**Figure 7:**
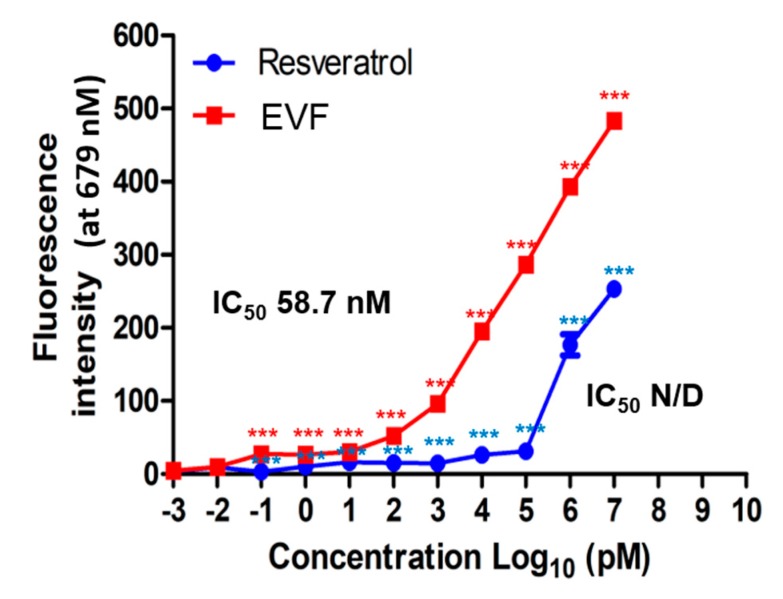
The effect of resveratrol and EVF on the in vitro activity of genotype 1b HCV NS3 helicase was determined by measuring the fluorescence intensity of Cy5-labeled double-stranded DNA at the excitation/emission wavelengths of 650/675 nm using increasing concentrations of vinifirin. The IC_50_ values were determined based on the corresponding response curve. A single asterisk (*) indicates that the *p*-value is between 0.1 and 0.5. A double asterisk (**) indicates that a *p*-value is between 0.1 and 0.01. A triple asterisk (***) indicates that the *p*-value is less than 0.01.

In previous studies, we have identified that resveratrol tetramers such as vitisin B can inhibit genotype 1b HCV NS3 helicase activity. Based on this observation, we hypothesized that vinifirin might inhibit NS3 helicase activity through its direct binding. To test this hypothesis, we utilized a GO-based NS3 helicase assay previously described [24]. In this assay, when Cy5-labelled double-stranded DNA is dissociated into single-stranded DNA by HCV NS3 helicase, the fluorescence of the released Cy5-labelled single-stranded DNA decreases by binding to GO. Therefore, blocking the HCV NS3 helicase with an inhibitor should lead to the restoration of fluorescent activity. As shown in Figure 7, EVF increased fluorescence in a dose-dependent manner at a concentration below its IC_50_ value (half maximal inhibitory concentration (IC_50_) = 58.7 nM), whereas the negative control resveratrol did not, up to a concentration of 10 μM. These data suggest that the inhibition of HCV NS3 helicase is a main antiviral mechanism of action by EVF.

### 3.5. The Pharmacokinetic Characterization of SVF in Mice

**Figure 8:**
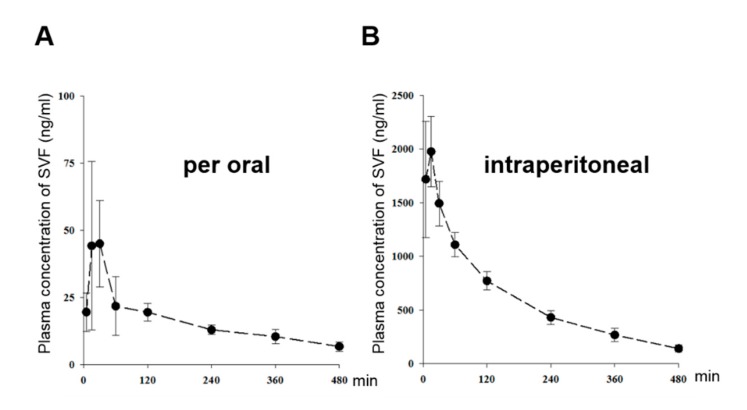
The time and mean plasma concentration relationship curves of SVF in mice after (**A**) oral administration (PO) of 50 mg/kg (●, *n* = 4) and (**B**) intraperitoneal administration (IP) of 15 mg/kg (●, *n* = 4). Vertical bars represent standard deviation.

In order to study the pharmacokinetic characteristics of SVF, we measured the plasma concentration of SVF after oral and intraperitoneal administration (Figure 8A,B). We observed plasma profile patterns and pharmacokinetic properties of SVF similar to those reported in our previous results [25]. Following an oral dose of 50 mg/kg, the mean Cmax of SVF was 53.5 ± 28.2 ng/mL occurring at a Tmax of 22.5 min (ranges, 15‒30 min), which indicates fast absorption (Figure 8A). Following an intraperitoneal dose of 15 mg/kg, plasma concentrations were much higher than those after oral dosing; the Cmax values were 2130 ± 403 ng/mL (Figure 8B). Previous studies have shown that the oral bioavailability of SVF is below 1% in mice [25]. These results suggest that its extremely low oral bioavailability might be due to very low permeability in the gastrointestinal tract and intestinal first-pass effects, but not due to hepatic first-pass effects. Intraperitoneal dosing might be a favorable route of administration for SVF.

## 4. Discussion

In this study, we prepared three different versions of viniferin (EVF, SVF-5Ac, and SVF) via plant-derived extraction and chemical synthesis (Figure 1). We identified all three as potential HCV replication inhibitors in 2a and 1b HCV genotype replicon cells (Figure 2, Figure 3 and Figure 4). We found that their inhibition of HCV genome replication led to reduced expression levels of viral proteins in a dose- and time-dependent manner (Figure 5 and Figure 6). In addition, we also confirmed that the main inhibitory mechanism of HCV replication by EVF was its suppression of HCV NS3 helicase activity (Figure 7). Finally, we found that intraperitoneal administration of SVF improved its pharmacokinetic properties when compared to oral administration in a mouse model (Figure 8).

EVF demonstrated 2.0- and 3.2-fold more antiviral activity than SVF in both genotype 2a renilla luciferase-based and RT-PCR-based HCV replication assays, respectively (Figure 2 and Figure 3). Its higher antiviral potency was also evident in genotype 2a NS3 Western blot analysis (Figure 5). Since SVF is a racemic mixture of (+)-ε-viniferin (EVF) and (-)- -viniferin, these data suggest the potential lack of antiviral activity of (-)-ε-viniferin. However, this structure and activity relationship does not seem to be applicable to genotype 1b HCV replication since SVF showed more potent antiviral activity than EVF in genotype 1b HCV replication (Figure 4). Interestingly, there was no difference in the NS3 protein reduction capacity of SVF and EVF (Figure 6). Based on these data, we conclude that an HCV genotype-specific difference in the antiviral activity of SVF and EVF appears to exist. With respect to the potential effects of penta-acetylation of viniferin on HCV replication, it seems to mediate a detrimental effect on HCV replication since its removal contributed to increased antiviral potency in the genotype 2a HCV reporter assay (Figure 2). This negative effect of penta-acetylation on HCV replication was also validated by the genotype 1b HCV RT-PCR-based replication assay (Figure 4). However, its removal resulted in decreased antiviral potency in the genotype 2a RT-PCR-based replication assay (Figure 3) and the genotype 2a NS3 Western blot-based assay (Figure 5). The presence of a renilla luciferase gene in genotype 2a HCV replicon may contribute to this discrepancy via an as yet unknown mechanism.

HCV helicase helps coordinate polyprotein translation and processing, and it can strip RNA-binding proteins from viral RNA, which aids in translation. The NS3 helicase is a Y-shaped protein composed of three domains. Domains 1 and 2 are RecA-like domains[29], the ATP-binding site is located between domains 1 and 2, and oligonucleotides are thought to be located between two RecA-like domains and domain 3 [30]. In this study, we confirmed that vinifirin displays different antiviral effects on different HCV genotypes. A difference in DNA unwinding rates between genotypes 1 and 2 HCV helicases has been reported [31]. The genotype 2a HCV helicase unwinds DNA faster than other HCV genotype helicases, possibly because it binds more tightly to target DNA. This might enhance the processivity of the helicase enzyme [32,33,34]. A more rapid helicase activity might be one of the reasons why genotype 2a HCV replication is more sensitive to viniferin than genotype 1b. Since we confirmed the inhibitory effect of SVF only on HCV genotype 1b helicase (Figure 7), it would be interesting to test the impact of SVF on HCV genotype 2a helicase in the future. These data could provide clues on the genotype-specific effects of the three viniferin versions on HCV replication.

Several biological functions of viniferin have been demonstrated. For example, viniferin exhibited hepatoprotective activity and had a potent inhibitory effect against lipoxygenase and the oxidation of low-density lipoprotein and high-density lipoprotein [35]. It was also shown to prevent inflammation [36]. Furthermore, vinifirin was even identified as an anti-Alzheimer’s agent. Indeed, it inhibits Aβ-induced neuronal apoptosis through regulation of the SIRT1-ROCK1 signaling pathway and Aβ aggregation [37]. However, these effects do not appear to be related to viniferin’s role in the inhibition of HCV replication considering its nano-molar rage of inhibitory activity against HCV NS3 helicase in vitro (Figure 7). However, a micro-molar range of antiviral potency in cell-based assays seems to be relatively high. This loss of antiviral potency could be due to its cell permeability and stability in the cells.

The concentration of viniferin in wine is between 0.1 and 4.3 mg/L [36]. Viniferin has been shown, in vitro, to possess antioxidant, anti-inflammatory, anti-carcinogenic, anti-viral, and cardioprotective activities. In vivo, the bioavailability of vinifirin is reported to be low, notably because of its rapid and intensive metabolic conversion [38]. After absorption, several forms of vinifirin were found in the bloodstream such as the native form and as glucuronide or sulfate metabolites. Glucuronidation and sulfation, which can be performed by UDP-glucuronosyltransferase (UGT) and sulfotransferase (SULT), respectively, are major metabolic pathways for numerous polyphenols including resveratrol [39,40]. Perhaps, for similar reasons described above, vinifirin has a low absorption rate in vivo [41]. In this report, the oral bioavailability of viniferin is usually less than 1%. Viniferin is intensely metabolized (i.e., 70–80% of the molecules are converted into conjugates) [42]. Thus, the oral bioavailability of vinifirin is extremely low, perhaps owing to its low absorption through the intestinal epithelium and to their intense metabolism [25]. In a previous study, we identified that vitisin B has a low EC_50_ value (0.006 μM) for inhibiting HCV replication in vitro [14]. Vinifirin also has an antiviral effect in vitro (EC_50_ value = 1 μM) [14]. However, vinifirin has a low level of pharmacokinetic dynamics through oral drug delivery systems. In Figure 8, we showed its improved bioavailability via intraperitoneal injection compared to oral administration. However, given that most HCV therapeutics are already developed as orally available forms, the pharmacokinetic behavior of viniferin needs to be further improved in order to make it a more attractive as a member of a new class of HCV drugs [43].

Th therapeutic efficacy of the recently developed direct-acting antivirals against HCV has been remarkable since approximately 90% of chronic HCV cases can be cleared with the new combination treatment regimen [13]. They include of glecaprevir (NS3/4A protease inhibitor)/pibrentasvir (NS5A inhibitor), sofosbuvir (NS5B RNA polymerase inhibitor)/ledipasvir (NS5A inhibitor), sofosbuvir/daclatasvir (NA5A inhibitor), sofosbuvir/simeprevir (NS3/4A protease inhibitor), sofosbuvir/velpatasvir (NS5A inhibitor), and elbasvir (NS5A inhibitor)/grazoprevir (NS3/4A protease inhibitor) [11]. Depending on the HCV genotype, the liver condition of the patient (cirrhosis), HIV coinfection status, and the patient’s ethnicity, a specific type, and duration of anti-HCV treatment plan vary [11]. However, in spite of the supreme antiviral efficacy and high treatment-success rate of current HCV drugs, these direct-acting antivirals cannot be free from a viral resistance problem due to the ever-evolving nature of the RNA virus [44]. Since all of the current anti-HCV drugs target either NS3/4A protease, NS5A protein, and NS5B RNA polymerase, development of a new class of anti-HCV drugs targeting other viral functions would be the best option for efficient suppression of the viral resistance. In this regards, HCV NS3 helicase inhibitors including vitisin B and viniferins would serve as another class of antiviral regimens reserved for current HCV drug-resistant patients.

Genetic structures and RNA sequences of other flaviviruses such as Dengue fever virus, Japanese encephalitis virus, Yellow fever virus, West Nile virus, and Zika virus were reported to be well conserved [45]. Especially, the relatively high sequence homology in the NS3 helicase region of flaviviruses raises the possibility of development of anti-NS3 helicase inhibitors as pan-flavivirus antiviral drugs. Given the unavailability of effective antiviral measures for most flavivirus infections except HCV, it would be imperative to test the effects of these three kinds of viniferins on viral helicase activity as well as the replication efficiency of other flaviviruses in the near future.

In summary, we successfully prepared three different versions of viniferin, including EVF, SVF-5Ac, and SVF. We confirmed their anti-HCV replication activities with minimal cytotoxicity. Anti-HCV NS3 helicase activity by EVF was also verified in vitro. Finally, we found that the intraperitoneal administration of SVF improved its pharmacokinetic properties. Overall, the antiviral and pharmacokinetic properties of these three viniferin versions warrant their further study as members of a promising new class of anti-HCV therapeutics.

## Figures and Tables

**Table 1:** Comparison of EC_50_, CC_50_, and T_1/2_ values for EVF, SVF-5Ac, and SVF based on their results from genotype 2a and 1b luciferase reporter assay, RT-PCR, and Western blot analyses.

Antiviral Assays Used	EVF			SVF-5Ac			SVF		
	EC_50_ (M)	CC_50_ (M)	T_1/2_ (h)	EC_50_ (M)	CC_50_ (M)	T_1/2_ (h)	EC_50_ (M)	CC_50_ (M)	T_1/2_ (h)
GT 2a luciferase reporter assay	0.1	>10	15.5	2.4	>10	12.6	0.2	>10	21.9
GT 2a RT-PCR	2.9	N/A	10	4.7	N/A	11.1	9.3	N/A	62.5
GT 1b RT-PCR	7	N/A	10.9	10.4	N/A	18.1	1.7	N/A	7.7
GT 2a Western blot	1.2	N/A	35.1	1	N/A	>72	6.4	N/A	37.1
GT 1b Western blot	0.9	N/A	58.2	0.8	N/A	29.6	0.9	N/A	13.1
